# Infection risk in cardiothoracic surgery due to contaminated heater cooler units

**DOI:** 10.1186/2047-2994-4-S1-P73

**Published:** 2015-06-16

**Authors:** T Götting, D Jonas, D Scheibert, S Klassen, W Ebner

**Affiliations:** 1 University Medical Center Freiburg, Freiburg, Germany

## Introduction

When several cases of severe bacterial infections with *Mycobacterium chimaera* in a Swiss hospital became public so called heater cooler units (HCU) used during open heart surgery were suspected to be the origin of the pathogens.

## Objectives

After confirming that the tanks of the HCU used in our facility were contaminated we attempted to identify potential transmission ways.

## Methods

To enable a timely risk assessment and due to the fact that *Mycobacterium chimaera* are very slow growing organisms we used non-fermenters as a surrogate parameter for water associated pathogens. In an experimental setting we conducted air sampling in different distances to the operating HCU. In addition air was sampled in the area of the operating table while HCU were placed at their usual location in the operating room. This was conducted either when HCU were operating or switched off. Air (200 L in 2 minutes) was collected by air sampler MBASS30LKS100 and conducted over microbiological plates for culturing.

## Results

Non-fermenters could be identified in up to three meters distance to the operating HCU. As long as the HCU were not operating cultures from the area of the operating table remained negative for non-fermenters. Once the HCU were started cultures showed considerable growth of non-fermenters.

## Conclusion

Considering our results pathogens from contaminated HCU tanks are disseminated via aerosol formation. Thus there is a potential way of transmission to patients undergoing open heart surgery. So far the extend of the problem is unknown which is why we would like to encourage other hospitals to initiate similar examinations. Our results suggest that HCU should be banned from the operating room. As they are also used in intensive care units this should be further evaluated as well.

## Disclosure of interest

None declared.

